# Homeoprotein SIX1 compromises antitumor immunity through TGF-β-mediated regulation of collagens

**DOI:** 10.1038/s41423-021-00800-x

**Published:** 2021-11-15

**Authors:** Wancheng Liu, Meiling Gao, Lili Li, Yu Chen, Huimin Fan, Qiaomei Cai, Yueyue Shi, Chaohu Pan, Junxiao Liu, Lucy S. Cheng, Heng Yang, Genhong Cheng

**Affiliations:** 1 Institute of Systems Medicine, Chinese Academy of Medical Sciences & Peking Union Medical College, Beijing, 100005 China; 2 Suzhou Institute of Systems Medicine, Suzhou, 215123 China; 3 Department of Dermatology, University of Pittsburgh Medical Center, 3708 Fifth Avenue, Suite 500.68, Pittsburgh, PA 15213 USA; 4 Department of Microbiology, Immunology & Molecular Genetics, University of California, Los Angeles, CA USA

**Keywords:** Homeoprotein SIX1, anti-tumor immunity, collagens., Tumour immunology, Cancer microenvironment

## Abstract

The tumor microenvironment (TME), including infiltrated immune cells, is known to play an important role in tumor growth; however, the mechanisms underlying tumor immunogenicity have not been fully elucidated. Here, we discovered an unexpected role for the transcription factor SIX1 in regulating the tumor immune microenvironment. Based on analyses of patient datasets, we found that SIX1 was upregulated in human tumor tissues and that its expression levels were negatively correlated with immune cell infiltration in the TME and the overall survival rates of cancer patients. Deletion of *Six1* in cancer cells significantly reduced tumor growth in an immune-dependent manner with enhanced antitumor immunity in the TME. Mechanistically, SIX1 was required for the expression of multiple collagen genes via the TGFBR2-dependent Smad2/3 activation pathway, and collagen deposition in the TME hampered immune cell infiltration and activation. Thus, our study uncovers a crucial role for SIX1 in modulating tumor immunogenicity and provides proof-of-concept evidence for targeting SIX1 in cancer immunotherapy.

## Background

Cancer is a global public health problem, and tumors develop in response to changes in both cancer cells and the tumor microenvironment (TME). Alterations in gene expression profiles and genomic mutations are necessary for normal cells to transform into cancer cells. Cancer cells within the TME can regulate the composition of the TME. On the other hand, the TME also plays crucial roles in tumor growth, therapeutic response, and patient outcomes [1]. There is growing evidence showing that altering gene expression in cancer cells can directly or indirectly affect the TME [2]. The transcription factor sine oculis homeobox 1 (SIX1), as a critical regulator of organogenesis, is highly expressed during embryonic development but is rarely expressed in normal human adult tissues [3]. Interestingly, SIX1 reappears in several human malignancies such as pancreatic cancer [4], breast cancer [5], and lung cancer [6]. However, the role of SIX1 in tumor growth and its associated mechanisms involved in regulating the TME remain to be elucidated.

The noncancer cells in the TME primarily consist of stromal cells (e.g., fibroblasts, pericytes, and mesenchymal stromal cells) and immune cells (including T and B lymphocytes, tumor-associated macrophages, and NK cells) [7]. Tumor-infiltrating lymphocytes (TILs) are immune cells that play pivotal roles in cancer initiation and progression [8]. Based on the infiltration and activity of antitumor T cells, the TME may be “noninflamed”, “cold T cell-infiltrated” or “hot T cell-infiltrated” [9]. Most cancers, such as sarcoma (SARC) and colon adenocarcinoma (COAD), are cold tumors with low immune cell infiltration and exhibit relatively low responsiveness to immune checkpoint inhibitors. In addition, the TME is also composed of an extracellular matrix (ECM), which is primarily regarded as the physical scaffold holding cells and tissues together. However, recent studies have shown that the ECM can also affect cancer cell adhesion, migration, and metastasis [10]. Collagen dominates the ECM as the most abundant component. Either increased or decreased deposition of collagen may influence immune cell infiltration and tumor progression [11].

Based on protein structure and localization, the collagen family can be divided into several groups [12]. Most collagen members are upregulated in various tumors [13], and they promote tumor initiation and progression. A high collagen density contributes to chemotherapy and immunotherapy resistance [14] and could be a new predictor of prognosis [15, 16,]. It has recently been reported that collagen density can regulate the activity of tumor-infiltrating T cells through the collagen receptor LAIR-1 [17, 18,]. Not only fibroblast-derived collagen but also cancer cell-derived collagen can affect the TME [19]. However, the mechanisms by which cancer cells affect tumor growth through collagen gene expression regulation in the TME are not clear.

Previous work has elucidated the roles of SIX1 in tumor metabolism, growth, and poor prognoses [20]. Nevertheless, the potential effects of SIX1 on the TME and the underlying mechanisms remain unknown. Using CRISPR/Cas9-mediated knockout of the *Six1* gene in cancer cells, we studied the impact of *Six1* deficiency on the triggering of antitumor immunity in the TME and explored the potential mechanisms both in vitro and in vivo.

## Results

### High expression of Six1 negatively correlates with immune cell infiltration in the TME and patient survival rates

**Fig. 1:**
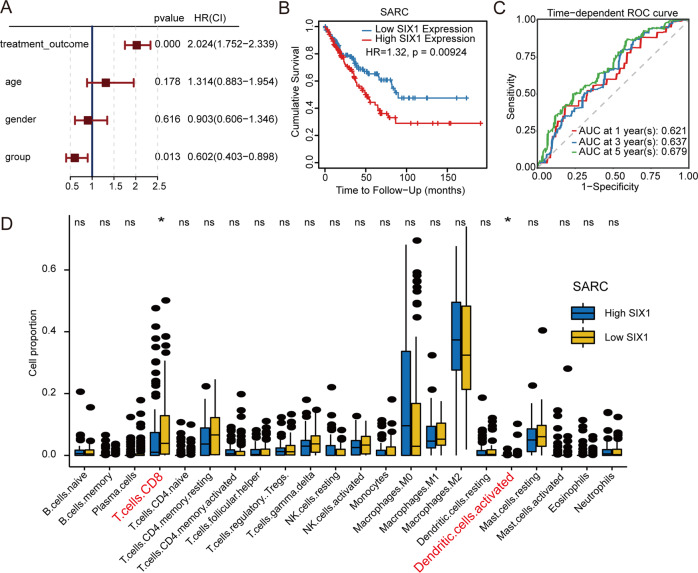
High expression of Six1 negatively correlates with immune cell infiltration in the TME and patient survival rates. **A** Prognostic value of the risk scores in univariate Cox regression models. **B** The clinical relevance of gene expression across SARC from the Gene_Outcome of Exploration module in TIMER2.0. Gene_Surv used a Cox proportional hazard model to evaluate the outcome significance of Six1 expression. **C** Time-dependent ROC curves for survival prediction with AUC values. **D** Comparison of the proportion of TICs between the high and low Six1 expression groups of SARC samples calculated by CIBERSORT. Boxplots display the proportions of 22 immune cells

In the course of investigating the molecular mechanisms responsible for regulating the TME through aberrant gene expression in cancer cells, we found that SIX1 was highly expressed in the cancerous tissues of most tumors (Fig. S1A). Sarcoma (SARC) usually caused by problems in human muscle refers to malignant tumors. A univariate analysis based on the expression levels of SIX1, treatment outcome, age, or sex (Table 1) showed that the expression levels of SIX1 (HR = 0.602, *P* = 0.013) and treatment outcome (HR = 2.024, *P* = 0.000) were independent prognostic factors for SARC patients (Fig. 1A). Clinically relevant studies on SIX1 expression across SARC performed with TIMER2.0 [21] showed that SARC patients with high SIX1 expression had worse overall survival (Fig. 1B). Moreover, the prognostic efficiency of SIX1 expression was assessed by time-dependent ROC curves, which showed that the area under the curve (AUC) values were 0.621 (1 year), 0.637 (3 years), and 0.679 (5 years) (Fig. 1C), indicating that SIX1 expression has good predictive value for the overall mortality of SARC patients. To further explore the relationship between the SIX1 expression levels and immune status in the TME, the proportions of immune cell types were obtained with CIBERSORT. Interestingly, cancerous tissues from SARC patients with lower expression of Six1 appeared to have higher proportions of both CD8 T cells and DCs (Fig. 1D), which was confirmed by analysis with TIMER2.0 (Fig. S1B). These results suggested that SIX1 might affect immune cell infiltration.

**Table 1:** Baseline characteristics of patients with sarcoma

Parameter	Subtype	Number of patients (%)
Treatment outcome	Complete response	126 (48.4)
(Chemotherapy)	Partial response	2 (0.7)
	Progressive disease	59 (22.6)
	Stable disease	12 (4.6)
	Unknown	61 (23.4)
Age	>60	130 (50)
	≤60	130 (50)
Sex	Female	142 (54.6)
	Male	118 (45.3)
Group	High SIX1 expression	130 (50)
	Low SIX1 expression	130 (50)

### Immune-dependent mechanisms are responsible for the reduced tumor growth of *Six1-*deficient cancer cells

**Fig. 2:**
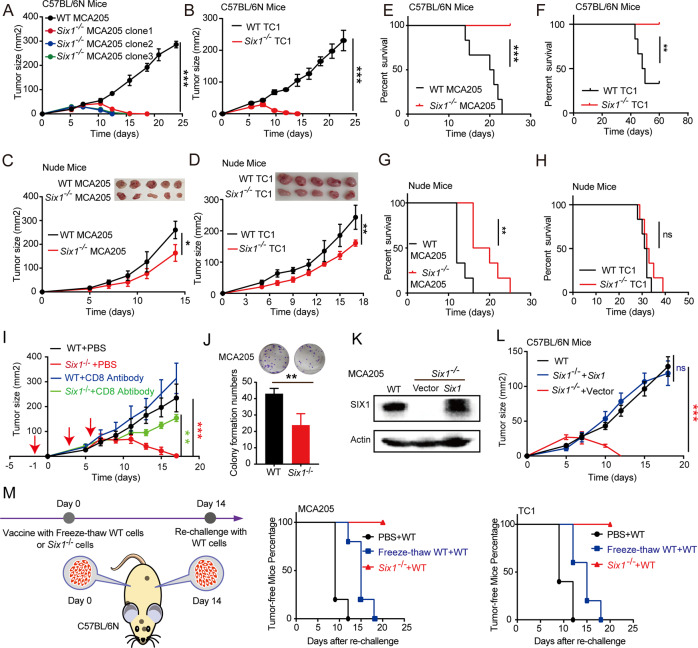
Immune-dependent mechanisms are responsible for the reduced tumor growth of *Six1-*deficient cancer cells. Tumor growth curves for C57BL/6N mice inoculated with MCA205 (**A**) or TC1 tumor cells (**B**). A total of 2 × 10^6^ *Six1*^*−/−*^ or WT tumor cells were subcutaneously transplanted into the back flank of C57BL/6N mice (*n* = 5), and tumor growth was monitored with calipers after the indicated time. Data are presented as the mean ± SD. ∗∗∗*p* < 0.001. Statistical significance was determined by the Mann–Whitney *U* test. Tumor growth curves for nude mice inoculated with MCA205 (**C**) or TC1 tumor cells (**D**). A total of 2 × 10^6^ *Six1*^*−/−*^ or WT tumor cells were subcutaneously transplanted into nude mice that lacked mature T lymphocytes. Tumor growth was monitored after the indicated times. Data are presented as the mean ± SD. ∗*p* < 0.05. Statistical significance was determined by the Mann–Whitney *U* test. **E** Kaplan–Meier survival curves for C57BL/6N mice injected with MCA205 tumor cells (*n* = 6 mice for each group). A total of 2 × 10^6^ *Six1*^*−/−*^ or WT MCA205 tumor cells were injected intravenously into C57BL/6N mice, and the number of dead mice was recorded every day. ∗∗∗*p* < 0.001, log-rank test. **F** Kaplan–Meier survival curves for C57BL/6N mice injected with TC1 tumor cells (*n* = 6 mice for each group). A total of 2 × 10^6^ *Six1*^*−/−*^ or WT TC1 tumor cells were injected intravenously into C57BL/6N mice. ∗∗*p* < 0.01, log-rank test. **G** Kaplan–Meier survival curves for nude mice injected with MCA205 tumor cells (*n* = 6 mice for each group). A total of 2 × 10^6^ *Six1*^*−/−*^ or WT MCA205 tumor cells were injected intravenously into nude mice. ∗∗*p* < 0.01, log-rank test. **H** Kaplan–Meier survival curves for nude mice injected with TC1 tumor cells (*n* = 6 mice for each group). A total of 2 × 10^6^ *Six1*^−/−^ or WT TC1 tumor cells were injected intravenously into nude mice. ns, not significant (*p* > 0.05, log-rank test). **I** C57BL/6N mice (*n* = 5/group) were inoculated subcutaneously with 2 × 10^6^ *Six1*^*−/−*^ or WT MCA205 tumor cells and treated intravenously with 200 µg/mouse anti-CD8 antibodies on days -1, 3, and 5. The red arrows indicate the time points for anti-CD8 antibody injection. Tumor growth was measured at the indicated time points starting on day 0. Data represent the mean ± SD. ∗∗*p* < 0.01, ∗∗∗*p* < 0.001. Statistical significance was determined by the Mann–Whitney *U* test. **J** Colony formation assay performed with *Six1*^*−/−*^ and WT MCA205 tumor cells. Data are represented as the mean ± SD. ∗∗*p* < 0.01, two-sided unpaired Student’s-test. **K** Western blot analysis of SIX1 in matched WT, *Six1*^*−/−*^ and *Six1*-restored MCA205 cells. **L** Tumor growth curves for C57BL/6N mice inoculated with WT, *Six1*^*−/−*^ or *Six1*-restored MCA205 cells. WT, *Six1*^*−/−*^ or *Six1*-restored MCA205 cells were subcutaneously transplanted into C57BL/6N mice, and tumor growth was monitored after the indicated time. Data are represented as the mean ± SD; *n* = 5 tumors for each group. ns, not significant; ∗∗∗*p* < 0.001; Mann–Whitney *U* test. **M** C57BL/6N mice were immunized subcutaneously in the left back flank with equal numbers of *Six1*^−/−^ tumor cells or freeze-thawed WT tumor cells (or PBS as a control). The freeze-thaw cycles were repeated three times. Fourteen days after immunization, live WT tumor cells were subcutaneously transplanted into the right back flank of the immunized mice. A schematic representation of the vaccination experiment with *Six1*^−/−^ tumor cells is shown in the left panel. Tumor growth was monitored after the indicated time. Data are represented as the tumor-free percentage; *n* = 5 tumors in each group

To determine the potential oncogenic role of SIX1 in SARC, a tumor xenograft model was used to investigate how SIX1 in cancer cells promotes tumor progression in vivo. *Six1*-deficient MCA205 mouse fibrosarcoma and TC1 mouse epithelial cell lines were generated with CRISPR/Cas9 technology using *Six1*-specific sgRNA pairs. WT cells transfected with an empty vector were used as controls. As both MCA205 cells and TC1 cells were derived from C57BL/6N mice, we established syngeneic tumor models using immunocompetent (C57BL/6N) mice transplanted with either WT or *Six1*^−/−^ tumor cells to visually observe the effects of *Six1* deficiency on tumor growth. All mice bearing WT MCA205 or TC1 cells had developed significant tumor masses at 14 days after transplantation, while none of the mice bearing *Six1*-deficient MCA205 or TC1 cells had (Figs. 2A, B and S2A, B), suggesting that *Six1* deficiency could prevent subcutaneous tumor growth in immunocompetent mice. To determine whether *Six1* deficiency could attenuate tumor growth in an immune-dependent manner, we also established a subcutaneous transplant tumor model with immunodeficient (nude) mice. The results showed that both WT cells and *Six1*^−/−^ cells developed into tumors in all transplanted nude mice (Fig. 2C, D), suggesting that *Six1* affected tumor growth in an immune-dependent manner. The same phenomenon was also observed with MC38 colon carcinoma cells (Fig. S2C).

To assess whether SIX1 affects tumor metastasis survival in an immune-dependent manner, *Six1*^−/−^ or WT tumor cells were inoculated into immunocompetent and immunodeficient mice by intravenous injection. All the C57BL/6N mice injected with *Six1*^−/−^ MCA205 or TC1 cells maintained metastasis-free survival between day 0 and day 60. In contrast, mice injected with WT MCA205 or TC1 cells exhibited 0 and 30% survival rates, respectively (Fig. 2E, F). On the other hand, all nude mice injected with either WT or *Six1*^−/−^ MCA205 or TC1 cells died by 40 days after injection (Fig. 2G, H). These results suggested that antitumor immunity might play a role in suppressing tumor growth and thereby prolonging the survival of immunocompetent mice transplanted with *Six1*^−/−^ cancer cells. Nude mice are immunocompromised due to their lack of mature T cells. To determine whether the reduced tumor growth of *Six1*-deficient cancer cells depends on CD8 T cells, an anti-CD8 antibody was administered by intravenous injection. As shown in the results (Fig. 2I), *Six1*^*−/−*^ MCA205 cells grew into solid tumors in mice injected with the anti-CD8 antibody.

Interestingly, tumors derived from WT cells grew slightly larger than those derived from *Six1*^−/−^ cells in nude mice at the late stage (Fig. 2C, D), suggesting that SIX1 may also have some potential intrinsic effects on tumor growth independent of the immune response. It has been reported that Six1 overexpression slightly increases cell proliferation [22]. We therefore performed a colony formation assay in vitro, and our results showed that *Six1*-deficient MCA205 cells had reduced colony formation compared to parental MCA205 cells (Fig. 2J). To avoid nonspecific effects of the CRISPR/Cas9 system, we also restored Six1 expression in the *Six1*^−/−^ MCA205 cell line, and the results showed that *Six1*-restored MCA205 cells grew tumors at a similar rate as parental MCA205 cells (Fig. 2K, L).

Given that *Six1* deletion suppressed tumor growth in a manner dependent on CD8^+^ T cells, which belong to the adaptive immune system, we hypothesized that *Six1*^−/−^ tumor cells, by triggering antitumor immunity, may protect the host from challenge with the corresponding WT tumor cells. To determine whether mice bearing *Six1*^−/−^ tumors develop antitumor immune memory, we immunized C57BL/6N mice with *Six1*-deficient cancer cells or freeze-thawed WT cancer cells subcutaneously on the left side and rechallenged the mice with live WT cancer cells on the right side after 2 weeks (Fig. 2M, left panel). Neither *Six1*^−/−^ nor freeze-thawed WT cancer cells developed into solid tumor masses on the left side. Interestingly, each mouse of immunization with *Six1*^−/−^ tumor cells completely inhibited WT tumor growth on the right side, and these mice were tumor free, whereas all the mice immunized with freeze-thawed WT tumor cells exhibited a much weaker effect (Fig. 2M). These observations suggested that animals immunized with *Six1*^−/−^ tumor cells established immunological memory, which effectively protected the host from challenge with the corresponding WT tumor cells.

### *Six1* deficiency triggers cellular immune responses in vivo

**Fig. 3:**
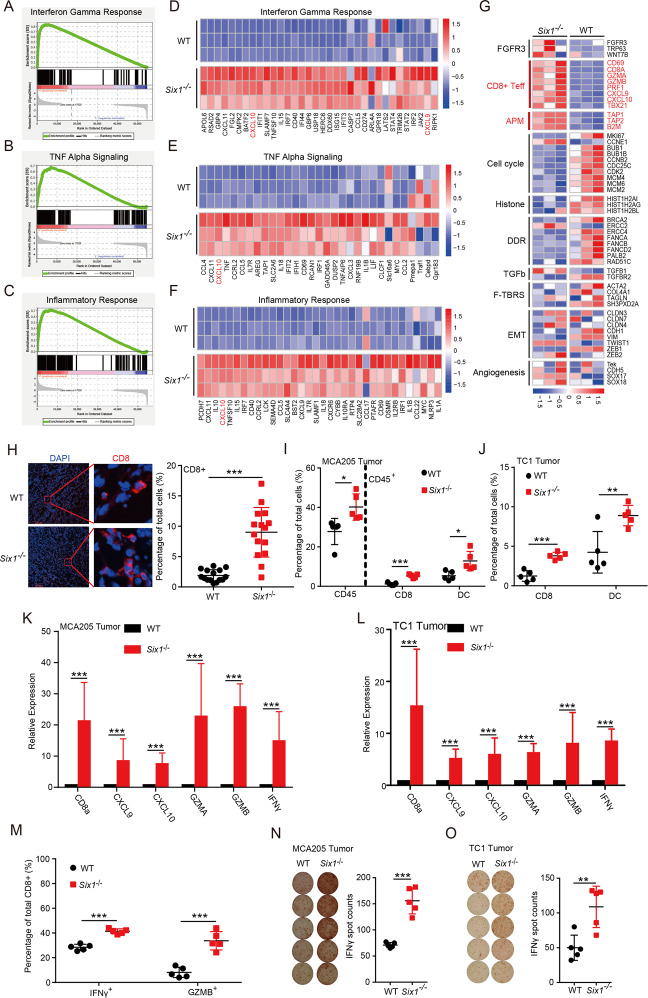
*Six1* deficiency triggers cellular immune responses in vivo. **A**–**C** GSEA of the differentially expressed genes between WT and *Six1*^*−/−*^ MCA205 tumors. Three positively regulated “hallmark” signatures are shown: **A** interferon-gamma response, **B** TNF alpha signaling, and **C** inflammatory response. The gene list was ranked with the signed (from log2-fold change [log2FC]) likelihood ratio for *Six1*^*−/−*^ versus WT MCA205 tumors. **D**–**F** Heatmaps for the normalized expression of transcripts from three positively regulated pathways (colors correspond to log2FC values). **G** Core biological pathways. The columns of the heatmap show gene expression grouped by pathway. **H** Representative CD8^+^ T cell staining of MCA205 tumor tissue. A total of 2 × 10^6^ *Six1*^−/−^ or WT MCA205 tumor cells were subcutaneously transplanted into C57BL/6N mice. On day 8, frozen sections generated from tumor tissues were subjected to immunostaining analysis for CD8^+^ T cells (red) and Hoechst staining for DNA (blue). CD8^+^ T cells were quantified by counting positive signals in 3 randomly selected fields (×20) for each tumor section using Image J, *n* = 5. Statistical comparisons were performed using an unpaired Student’s *t*-test, ***p < 0.001. Scale bar, 200 μm. FACS analysis of the proportions of major immune cell populations in MCA205 (**I**) and TC1 tumors (**J**). A total of 2 × 10^6^ *Six1*^−/−^ or WT MCA205 (**I**) or TC1 (**J**) tumor cells were subcutaneously transplanted into C57BL/6N mice. On day 8, tumor tissues were subjected to flow cytometric analysis. Data are shown as the mean ± SD for one of three independent experiments run with five replicates. *p < 0.05, **p < 0.01, ***p < 0.001 (unpaired Student’s *t*-test). RT–PCR analysis of the mRNA expression levels of the CD8a, Cxcl19, Cxcl10, GZMA, GZMB, and IFN-γ genes in MCA205 (**K**) and TC1 (**L**) tumor tissue samples. Tumor tissues from C57BL/6N mice transplanted as in **I** were subjected to RT–qPCR analysis. The data are presented as fold changes compared with WT tumors. Data are shown as the mean ± SD; ***p < 0.001, unpaired Student’s *t*-test. **M** FACS analysis of the proportions of major immune cell populations in MCA205 tumors. Tumor tissues from C57BL/6N mice transplanted as in **I** were subjected to flow cytometric analysis. CD8^+^IFN-γ^+^ and CD8^+^GZMB^+^ T cells are presented. Data are shown as the mean ± SD. ***p < 0.001 (unpaired Student’s *t*-test). ELISpot assay for the secretion of IFN-γ in MCA205 (**N**) and TC1 (**O**) tumors. Tumor tissues from C57BL/6N mice transplanted as in **I** were subjected to ELISpot analysis. The number of spots was enumerated on an ELISpot reader, and the results are presented as spot-forming units. Data are shown as the mean ± SD; **p < 0.01, ***p < 0.001, unpaired Student’s *t*-test

To understand how *Six1* deletion in cancer cells triggers an adaptive immune response to limit tumor growth, we performed transcriptomic RNA-sequencing (RNA-seq) analysis of WT and *Six1*^*−/−*^ MCA205 tumor tissue samples  (GSE183580). Expression analysis by GSEA revealed that three gene sets (“hallmark” signatures) including “Interferon-g (IFN-γ) response” (Fig. 3A), “Tumor necrosis factor-a (TNF-α) signaling” (Fig. 3B), and “Inflammatory response” were upregulated in *Six1*^*−/−*^ tumor tissues compared with WT MCA205 tumor tissues (Fig. 3C) [23]. Heatmaps for the most differentially expressed genes (DEGs) in these signatures between WT and *Six1*^*−/−*^ MCA205 tumors showed increased expression of numerous proinflammatory cytokines and chemokines, including TNF-α pathway components and C-X-C motif chemokine ligands 9 and 10 (Cxcl9 and Cxcl10) (Fig. 3D–F). It has been reported that TNF-α signaling plays an essential role in dendritic cell maturation [24, 25,], while CXCL9 and CXCL10 bind CXCR3 to induce the migration of activated T cells in vivo [26]. In the TME, these secreted factors can potentially enhance tumor antigen presentation and antitumor CD8^+^ T cell responses [27]. Moreover, RNA-seq data revealed that genes directly associated with the antigen presentation machinery and CD8^+^ T_eff_ signature were also increased in *Six1*^*−/−*^ tumor tissues (Fig. 3G) [28]. These results indicated that SIX1 expressed in cancer cells might play an important role in controlling immune responses.

As an indication of active adaptive immune responses, *Six1*-deficient tumor tissues exhibited massive infiltration of CD8^+^ T cells and DCs (Figs. 3H–J and S3A). In addition, increased expression of CD8α, CXCL9, CXCL10, Granzyme A (GZMA), Granzyme B (GZMB), and IFN-γ was observed in *Six1*-deficient tumor tissues (Fig. 3K, L). Higher percentages of CD8^+^ T cells isolated from *Six1*^*−/−*^ tumor tissues expressed effector T cell activation markers such as IFN-γ and GZMB (Figs. 3M and S3C), which was confirmed by an IFN-γ ELISpot assay (Figs. 3N, O and S3D). Collectively, these data suggested that cellular immune responses, particularly CD8^+^ T cell responses, were activated in TMEs containing *Six1*-deficient cancer cells.

### SIX1 promotes the expression of collagen genes

**Fig. 4:**
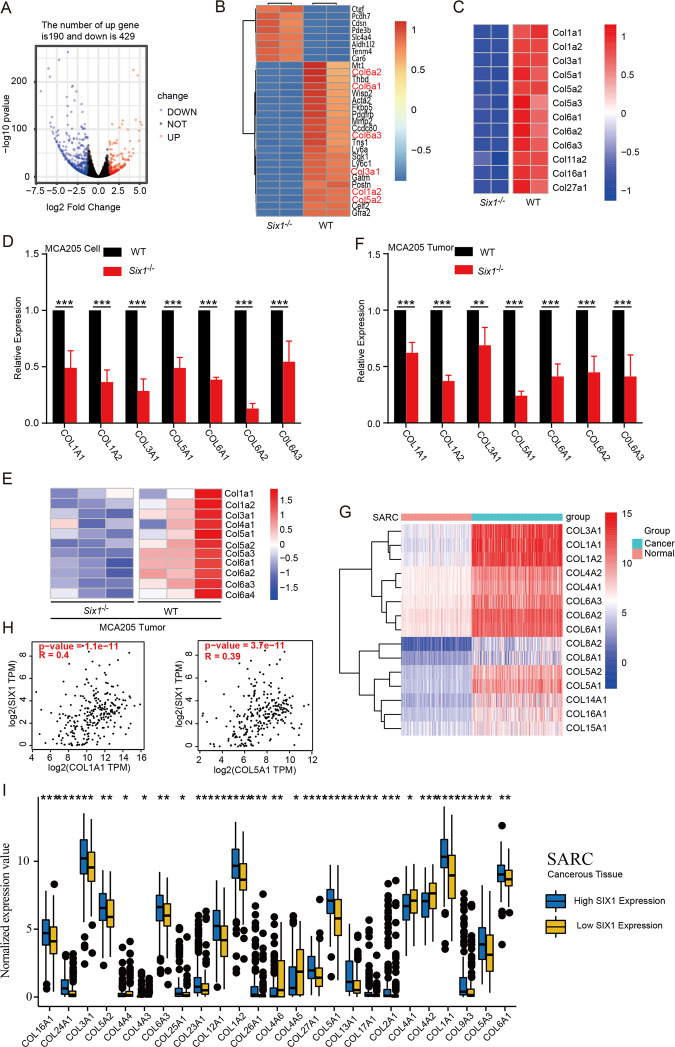
SIX1 promotes the expression of collagen genes. **A** Volcano plot showing the DEG expression profile between *Six1*^*−/−*^ and WT MCA205 cells using RNA-seq data, with the *x*-axis representing the fold change in gene expression and the *y*-axis representing *p* values. **B** Heatmap depicting the expression patterns of the top 30 DEGs between *Six1*^*−/−*^ and WT MCA205 cells with the smallest *p* values and most significant fold changes. Collagen genes are highlighted with red letters. **C** Heatmap depicting differential collagen gene expression patterns between *Six1*^*−/−*^ and WT MCA205 cells. **D** RT–qPCR analysis of the mRNA expression levels of collagen genes between *Six1*^*−/−*^ and WT MCA205 cells. GAPDH was used as a loading control; ***p < 0.001, unpaired Student’s *t*-test. **E** Heatmap depicting differential collagen gene expression patterns between *Six1*^*−/−*^ and WT MCA205 tumor tissues. Mice subcutaneously implanted with *Six1*^*−/−*^ or WT MCA205 cells (2 × 10^6^) were sacrificed by carbon dioxide asphyxiation on day 8 postimplantation, and tumors were harvested for RNA sequencing. **F** RT–qPCR analysis of the mRNA expression levels of collagen genes between *Six1*^*−/−*^ and WT MCA205 tissues. C57BL/6N mice were subcutaneously transplanted with 2 × 10^6^ *Six1*^*−/−*^ or WT MCA205 tumor cells, and the expression levels of collagen genes in tumor tissues were determined by RT–qPCR 8 days after transplantation. GAPDH was used as a loading control; ***p < 0.001, unpaired Student’s *t*-test. **G** Heatmap depicting the differential expression pattern of collagen genes between normal and cancerous tissue in SARC. A total of 260 SARC tumor samples were downloaded from the UCSC Xena database, and 200 normal muscle tissue samples with RNA-sequencing data were obtained from the Genotype-Tissue Expression (GTEx) database. Differentially expressed collagen genes between the SARC samples and normal controls were identified using the “limma” package. Genes with a log2 |fold change | >1 and false discovery rate (FDR) < 0.05 were considered DEGs. **H** The correlations between SIX1 and COL1A1 or COL5A1 transcripts in SARC cancer tissues were analyzed with GEPIA (http://gepia.cancer-pku.cn). **I** Comparison of the expression levels of collagen genes between high and low Six1 expression groups of SARC samples from the TCGA

Accumulating evidence indicates that cancer cell-intrinsic alterations can induce an effective immune response in the TME [29–31]. To further understand the molecular mechanisms by which *Six1-*deficient cancer cells trigger T cell activation in the TME, we performed another RNA-Seq analysis to identify DEGs between WT and *Six1*^*−/−*^ MCA205 cells (GSE183580). The results identified 619 DEGs including 190 upregulated genes and 429 downregulated genes (Fig. 4A). Among the top 30 DEGs shown in the heatmap, multiple collagen genes, including Col1a2, Col3a1, Col5a2, Col6a1, Col6a2, and Col6a3, were downregulated in *Six1*^*−/−*^ MCA205 cells (Fig. 4B, C), which was verified by RT–qPCR (Figs. 4D and S4A). Additionally, RNA-seq and RT–qPCR analyses of MCA205 tumor tissues showed similar results (Fig. 4E, F). These results therefore suggested that SIX1 might be a master regulator of collagen genes.

The collagen family, which includes 28 types with different α chains encoded by more than 40 genes, contains the most abundant ECM proteins [32], and more than 2/3 of collagen family genes are upregulated in tumor tissues [13, 33,]. We further analyzed the relationship between SIX1 and collagen expression based on the clinical data. Due to the lack of normal SARC samples in the TCGA database, we used muscle tissue data downloaded from the Genotype-Tissue Expression (GTEx) database as a normal control and found that numerous collagen genes were significantly upregulated in tumor tissue (Figs. 4G and S4B). Correlation analysis between *Six1* and several collagen genes performed with the GEPIA website [34] revealed statistically significant positive correlations between SIX1 and multiple collagen genes in SARC and COAD (Figs. 4H and S4C, D). Moreover, SARC and COAD tumor tissues were divided into two groups according to the expression level of SIX1. Most collagen genes were upregulated in the high Six1 expression groups (Figs. 4I and S4E). As SIX1 is rarely expressed in adult tissues [3], our studies suggested the possibility that aberrant expression of the *Six1* gene in tumor tissue contributed to tumor growth through collagen accumulation in the TME, leading to suppression of antitumor immunity.

### Reduced tumor growth of *Col6a1*-deficient cancer cells correlates with enhanced immune cell infiltration and activation

Increasing evidence indicates that a high-density collagen matrix reduces CD8^+^ T cell abundance [17] and that tumor-expressed collagens can suppress the immune response via the collagen receptor LAIR-1 [35, 36,]. This prompted us to investigate whether SIX1 inhibits the antitumor immune response by inducing collagen accumulation in the TME. Among the multiple collagen genes downregulated in *Six1*^*−/−*^ cells, collagen VI was expressed at the highest levels (Fig. S5A). Hence, we chose collagen VI as an example and investigated whether collagen VI secreted by cancer cells could suppress the antitumor immune response in the TME.

**Fig. 5:**
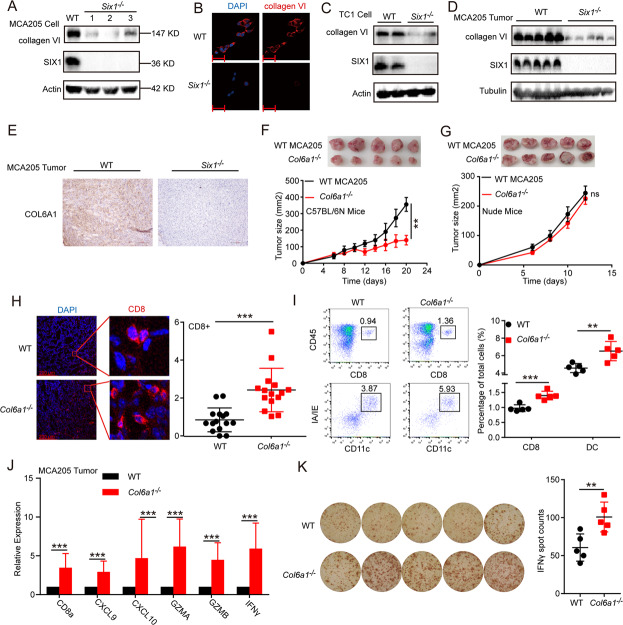
Reduced tumor growth of *Col6a1*-deficient cancer cells correlated with enhanced immune cell infiltration and activation. **A** Western blot analysis of COL6A1 and SIX1 in matched WT and *Six1*^*−/−*^ MCA205 cells. β-actin was used as a loading control. **B** Immunofluorescence staining analysis of collagen VI in WT MCA205 and *Six1*^−/−^ MCA205 cells. Scale bar, 50 μm. **C** Western blot analysis of COL6A1 and SIX1 in matched WT and *Six1*^*−/−*^ TC1 cells. β-actin was used as a loading control. **D** Western blot analysis of COL6A1 and SIX1 in WT and *Six1*^*−/−*^ MCA205 tumor tissues. A total of 2 × 10^6^ *Six1*^*−/−*^ or WT MCA205 tumor cells were subcutaneously transplanted into the back flank of C57BL/6N mice, and then tumor tissues were subjected to western blot analysis with antibodies specific for the indicated proteins 8 days after transplantation. β-actin was used as a loading control. **E** IHC analysis (original magnification, ×20) of the protein levels of COL6A1 in WT and *Six1*^*−/−*^ MCA205 tumor tissues. Tumor tissues from C57BL/6N mice transplanted as in **C** were embedded in paraffin and then subjected to IHC analysis. **F** Tumor growth curves for C57BL/6N mice inoculated with *Col6a1*^*−/−*^ or WT MCA205 tumor cells. A total of 2 × 10^6^ *Col6a1*^*−/−*^ or WT MCA205 tumor cells were subcutaneously transplanted into the back flank of C57BL/6N mice (*n* = 5), and tumor growth was monitored with calipers after the indicated times. Data are presented as the mean ± SD. ∗∗*p* < 0.01; statistical significance was determined by the Mann–Whitney *U* test. **G** Tumor growth curves for nude mice inoculated with *Col6a1*^*−/−*^ or WT MCA205 tumor cells. A total of 2 × 10^6^ *Col6a1*^*−/−*^ or WT MCA205 tumor cells were subcutaneously transplanted into the back flank of nude mice as in **F**. ns not significant; Mann–Whitney *U* test. **H** Representative CD8^+^ T cell staining of MCA205 tumor tissues. A total of 2 × 10^6^ *Col6a1*^*−/−*^ or WT MCA205 tumor cells were subcutaneously transplanted into the back flank of C57BL/6N mice. On day 12, frozen sections generated from tumor tissues were subjected to immunostaining analysis of CD8^+^ T (red) and Hoechst staining for DNA (blue). CD8^+^ T cells were quantified by counting positive signals in 3 randomly selected fields (×20) for each tumor section using Image J, *n* = 5. Statistical comparisons were performed using an unpaired Student’s *t*-test, ***p < 0.001. Scale bar, 200 μm. **I** Profiling of immune cells (defined by specific markers) in the TME by flow cytometry. A total of 2 × 10^6^ *Col6a1*^*−/−*^ or WT MCA205 tumor cells were subcutaneously transplanted into the back flank of C57BL/6N mice. On day 12, tumor tissues were subjected to flow cytometric analysis. Data are shown as the mean ± SD, *n* = 5. **p < 0.01, ***p < 0.001, unpaired Student’s *t*-test. **J** RT–qPCR analysis of CD8a, Cxcl9, Cxcl10, GZMA, GZMB, and IFN-γ mRNA expression levels in MCA205 tumor tissues. Tumor tissues from C57BL/6N mice transplanted as in **H** were subjected to RT–qPCR analysis. The data are presented as fold changes compared with WT tumors. Data are shown as the mean ± SD; ***p < 0.001, unpaired Student’s *t*-test. **K** ELISpot assay analyzing the secretion of IFN-γ in MCA205 tumor tissues. Tumor tissues from C57BL/6N mice transplanted as in **I** were subjected to ELISpot analysis. The number of spots was enumerated on an ELISpot reader, and the results are presented as spot-forming units. Data are shown as the mean ± SD; **p < 0.01, unpaired Student’s *t*-test

First, we found that the expression levels of COL6A1 were negatively correlated with the numbers of infiltrating CD8^+^ T cells in SARC and COAD patients with TIMER2.0 (Fig. S5B, C) [21, 37,]. In vitro, western blot analysis showed that collagen VI was downregulated in *Six1*-deficient MCA205 cells (Fig. 5A). The changes in collagen VI secretion/deposition outside cancer cells were examined by immunofluorescence staining. The results showed that while a large amount of collagen VI was deposited outside WT cancer cells, significantly reduced collagen deposition was observed around *Six1*-deficient cancer cells (Fig. 5B). Similar phenomena were observed for TC1 cells (Fig. 5C) and HepG2 cells (human hepatocellular carcinoma cells) (Fig. S5D). Western blot analysis also showed that collagen VI was downregulated in *Six1*-deficient MCA205 tumor tissues in vivo (Fig. 5D). We also performed immunochemistry (IHC) analysis and confirmed that COL6A1 levels were much lower in *Six1*^*−/−*^ MCA205 tumor tissues than in WT tumor tissues (Fig. 5E). Next, we explored whether COL6A1 influences the immune response in vivo. WT and *Col6a1*^*−/−*^ MCA205 cancer cells were subcutaneously transplanted into C576BL/6N or nude mice. WT MCA205 tumors were significantly larger than *Col6a1*^*−/−*^ MCA205 tumors in C576BL/6N mice (Fig. 5F), while there were no significant differences in nude mice (Fig. 5G). In addition, we observed enhancement of not only CD8^+^ T cell infiltration but also DC infiltration (Fig. 5H, I) and upregulation of CXCL9, CXCL10, GZMA, GZMB, and IFN-γ in *Col6a1*^*−/−*^ tumor tissues compared with WT MCA205 tumor tissues (Fig. 5J, K). These results are consistent with previous reports showing that tumors with decreased collagen levels have increased CD8^+^ TIL levels [36] and that a high-density collagen matrix reduces T cell proliferation and cytotoxic activity [17, 38,]. These results together indicated that *Col6a1* deficiency in cancer cells enhanced immune cell infiltration and activation in the TME.

We further explored the effect of collagen VI overexpression in *Six1*^*−/−*^ cancer cells on tumor growth. The results showed that CD8^+^ T cell infiltration was significantly reduced in the TME of *Six1*^−/−^ MCA205 tumors overexpressing *Col6a1* compared with that of tumors derived from vector control-treated cells (Fig. S5E, F), indicating that overexpression of collagen VI was sufficient to suppress the enhanced CD8^+^ T cell infiltration in *Six1*^−/−^ MCA205 tumors.

### SIX1 enhances the TGF-β signaling pathway by upregulating *Tgfbr2* expression

**Fig. 6:**
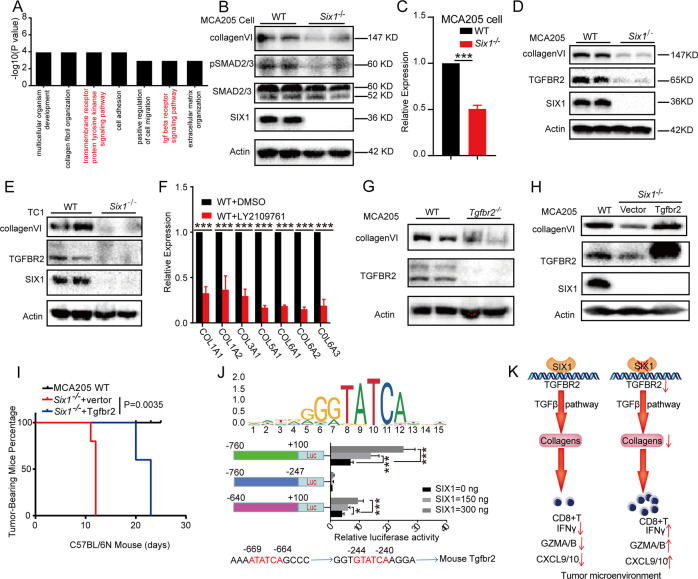
SIX1 enhances the TGF-β signaling pathway by upregulating *Tgfbr2* expression. **A** Gene Ontology (GO) enrichment analysis of DEGs between *Six1*^*−/−*^ and WT MCA205 cells. **B** Western blot analysis of COL6A1, pSMAD2/3, and SMAD2/3 in matched WT and *Six1*^*−/−*^ MCA205 cells. β-Actin was used as a loading control. **C** mRNA expression levels of *Tgfbr2* in matched WT and *Six1*^*−/−*^ MCA205 cells normalized to GAPDH; ***p < 0.001, unpaired Student’s *t*-test. **D** Analysis of TGFBR2 and COL6A1 protein levels in WT and *Six1*^*−/−*^ MCA205 cells using western blotting. β-actin was used as a loading control. **E** Analysis of TGFBR2 and COL6A1 protein levels in WT and *Six1*^*−/−*^ TC1 cells using western blotting. β-actin was used as a loading control. **F** Effects of LY2109761 on the mRNA expression levels of collagen genes in WT MCA205 cells. MCA205 cells were treated with LY2109761 at a dose of 10 μM for 48 h, and the mRNA expression levels of collagen genes were detected by RT–qPCR, with normalization to GAPDH; ***p < 0.001, unpaired Student’s *t*-test. **G** Western blot analysis of COL6A1 in WT and *Tgfbr2*^*−/−*^ MCA205 cells. β-actin was used as a loading control. **H** Western blot analysis of COL6A1, TGFBR2, and SIX1 in matched WT, *Six1*^*−/−*^ and *Six1*^*−/−*^ *Tgfbr2*-rescued MCA205 cell lines. β-actin was used as a loading control. **I** Percentages of tumor-bearing mice for C57BL/6N mice inoculated with WT, *Six1*^*−/−*^ or *Six1*^*−/−*^ *Tgfbr2*-rescued MCA205 tumor cells. A total of 2 × 10^6^ *Six1*^*−/−*^ or *Six1*^*−/−*^ *Tgfbr2*-rescued MCA205 tumor cells were subcutaneously transplanted into the back flank of C57BL/6N mice (*n* = 5), and tumor growth was monitored after the indicated times. The vertical axis represents the tumor-bearing mouse percentage, **J** Effect of SIX1 on PGL4.0-Tgfbr2 promoter. MCA205 cells were cultured in 24-well plates and transfected using Lipofectamine 3000 reagent according to the manufacturer’s instructions. Briefly, 100 ng/well PGL4.0-Tgfbr2 and 0, 150, or 300 ng/well VP64-Six1 or empty vector were cotransfected, and Renilla luciferase plasmids (30 ng/well) were also cotransfected as a normalization control for transcription efficiency. Luciferase activity was measured after 24 h of transfection. The results are expressed as relative luciferase activity (ratio of the luciferase activities versus the corresponding Renilla activity). Data are presented as the mean ± SD. *P < 0.05, **P < 0.01, ***P < 0.001. **K** Proposed model for collagen secretion from cancer cells upregulated by SIX1 to hamper antitumor immune responses

There is compelling evidence that the TGF-β pathway plays an important role in collagen production [39]. Active TGFβ binds to the cognate TGFβ receptor II and I (TGFβRII and TGFβRI) complex, initiating Ser/Thr phosphorylation of intracellular downstream Smad2/3 and subsequently leading to transcriptional activation of target genes. Gene expression analysis comparing *Six1*^*−/−*^ and WT MCA205 cells using the DAVID database [40] revealed subsets of DEGs involved in “collagen fibril organization,” “cell adhesion,” and “transforming growth factor-beta receptor signaling pathway,” (Fig. 6A), indicating the potential of SIX1 to act as link between the TGF-β pathway and collagen gene expression. Indeed, the levels of phospho-smad2/3 were reduced in *Six1* knockout MCA205 cells compared with the corresponding WT cells, while total smad2/3 levels showed no significant change (Fig. 6B). It was reported that inhibition of mouse TGFBR2 blunts collagen deposition [41], and *Col6a1* was identified as a TGF-β/Smad3 target [42]. RT–PCR analysis showed that the mRNA expression levels of *Tgfbr2* were lower in *Six1*^*−/−*^ MCA205 cells than in WT MCA205 cells (Fig. 6C). Western blot analysis also showed that the protein levels of TGFBR2 and COL6A1 were significantly reduced in *Six1*^*−/−*^ MCA205 cells compared to WT MCA205 cells (Fig. 6D, E). We further verified that collagen levels were markedly reduced after inhibition of TGFBR2 with the novel TGF-βR inhibitor LY2109761 [43] or Cas9-mediated knockout of *Tgfbr2* (Fig. 6F, G). Interestingly, the COL6A1 protein in *Six1*^*−/−*^ MCA205 cells was restored to levels similar to those in WT MCA205 cells by exogenous TGFBR2 overexpression (Fig. 6H). More importantly, TGFBR2-overexpressing *Six1*^*−/−*^ MCA205 cells not only developed into tumors but also produced tumors that were maintained in C57BL/6 mice for a much longer period of time than those produced from *Six1*^*−/−*^ MCA205 cells (Fig. 6I). Interestingly, the tumors that developed from TGFBR2-overexpressing *Six1*^*−/−*^ cells eventually regressed over 3 weeks. These data suggested that SIX1 might control tumor growth at least partially through TGFBR2.

SIX1 acts as a transcription factor, and the JASPAR CORE database [44] revealed that the SIX1 binding DNA motif contains a TATCA sequence (Fig. 6J). To test whether SIX1 transcriptionally regulates *Tgfbr2* gene expression, we examined ~2 kb of the *Tgfbr2* promoter region for putative SIX1-binding sites. We found that the *Tgfbr2* gene contained two putative SIX1-binding sites located at positions −240 to −244 (motif A) and −664 to −669 (motif B) relative to the transcriptional start site. We subsequently constructed three luciferase reporters driven by the *Tgfbr2* gene promoter containing the different putative SIX1-binding sites. A dual-luciferase reporter assay was performed to estimate promoter activity, and the results showed that *Six1* overexpression significantly increased the *Tgfbr2* promoter activity in cells transfected with the promoter reporters containing either motif A or motif A + B but not motif B only (Fig. 6J). Furthermore, relative luciferase activity increased as the concentration of *Six1* increased, suggesting that SIX1 increases *Tgfbr2* gene transcription by binding to motif A in the promoter region of the *Tgfbr2* gene. We also selected a SIX1-Flag-expressing stable MCA205 cell line for ChIP-qPCR analysis since anti-SIX1 antibody cannot be used for chromatin immunoprecipitation (ChIP) analysis (Fig. S6A). The results showed that anti-Flag antibody enriched DNA fragments including position –240 but not position 664 (Fig. S6B). In addition, TGF-β1 strongly activated SMAD2/3 phosphorylation and collagen VI expression in WT MCA205 cells but not in *Six1*^−/−^ MCA205 cells (Fig. S6C), indicating that SIX1 may bind to position –240 in the *Tgfbr2* promoter region to drive *Tgfbr2* transcription.

Overall, our studies identified a novel pathway by which abnormal expression of SIX1 in cancer cells promotes tumor growth through upregulation of TGFBR2 and collagen to suppress immune cell infiltration and activation in the TME. A proposed model for collagen secretion by cancer cells upregulated by SIX1 to hamper antitumor immune responses is shown in Fig. 6K.

## Discussion

Numerous studies have demonstrated the critical roles of the TME in both cancer development and treatment [45, 46,]. Tumors with a T cell-inflamed TME are often referred to as “hot” tumors. Patients with “hot” tumors have excellent responses to treatment and a good prognosis. Conversely, tumors lacking T cell infiltration are called immunological deserts or “cold” tumors, such as SARC and COAD, and are often associated with a poor prognosis. Matrix features in the TME, such as relatively high collagen deposition, can suppress T cell infiltration. However, it is not yet fully understood how cancer cells alter the ECM to actively suppress T cell infiltration and activation. In this study, we found that SIX1 was upregulated in various cancer cells (Fig. S1A) and that SIX1 levels were negatively correlated with poor survival rates and immune cell infiltration in SARC (Fig. 1). Deletion of *Six1* in cancer cells strongly inhibited tumor growth in C57BL/6N mice by inducing an adaptive immune response. Furthermore, our results showed that SIX1 promoted the expression of collagen genes in cancer cells, leading to collagen deposition in the ECM, which subsequently suppressed immune cell infiltration and activation in the TME. Therefore, our findings provide evidence explaining how abnormal expression of *Six1* in cancer cells affects antitumor immune responses by reshaping the TME.

Previous studies have shown that SIX1 promotes tumor lymphangiogenesis, angiogenesis, growth, and metastasis [22, 47,]. We also found that *Six1*-deficient cancer cells developed into tumors only in immunodeficient mice, not in immunocompetent mice (Fig. 2A–D). These results indicate the critical role of SIX1 in regulating tumor growth through immune-dependent mechanisms. Indeed, RNA-seq analysis of tumor tissues showed that antitumor immune response genes were expressed at higher levels in tumors developed from *Six1*^−/−^ cells than those developed from *Six1*^+/+^ cancer cells (Fig. 3A–G). This is consistent with our observations by flow cytometry and immunofluorescence staining that tumors developed from *Six1*^−/−^ cancer cells had increased DC and CD8^+^ T cell infiltration (Fig. 3H–J). In our study, CD8^+^ T cells in the TME of *Six1*^−/−^ tumors produced multiple effector cytokines, such IFN-γ and GZMB, indicative of cellular immune responses (Fig. 3M). Moreover, CD8-blocking antibodies enhanced the tumor growth of *Six1*^−/−^ cancer cells (Fig. 2I). Together, the data demonstrate that *Six1* deletion in cancer cells enhances antitumor immune responses and especially stimulates cellular immune responses.

SIX1 can alter the expression of a broad array of cytokines in different contexts (either negatively or positively) [22, 48,] by binding to a subset of inflammatory gene promoters [49]. Therefore, while cytokines regulated by SIX1 have been reported, whether other factors induced by SIX1 are involved in the regulation of antitumor immune responses remains to be elucidated. Interestingly, analysis of cell RNA-seq data showed that the loss of *Six1* was correlated with the downregulation of multiple collagen genes (Fig. 4B), which was further validated by RT–qPCR (Fig. 4D). Fibroblasts and myofibroblasts are normally the major sources of collagen, which acts as the scaffold in the TME and is thought to be a passive player during tumor progression. Recently, tumor-expressed collagens have been shown to inhibit antitumor immune responses by blocking immune cell infiltration and T cell activation via LAIRs, which are highly expressed on T cells [50]. However, David et al. recently reported that PD-(L)1 blockade was associated with increased intratumoral collagen deposition, especially collagen VI deposition [36]. Through establishing high or low collagen-expressing cell lines, they confirmed that tumors with low collagen levels had higher CD8^+^ TIL levels. Consistently, we found that tumor tissues developed from *Six1*-deficient cells compared with those developed from the corresponding parental cancer cells had notably reduced expression of multiple collagen genes (Fig. 4F, E). Our mechanistic data were further strengthened by analyses of sarcoma patient datasets, in which most collagen members were upregulated in cancerous tissue compared with normal tissue (Fig. 4G) and were positively correlated with the expression levels of Six1 (Figs. 4I and S4D). Sarcomas represent numerous very different tumor types, such as fibrosarcoma, osteosarcoma, and rhabdomyosarcoma. In our study, 260 SARC tumor samples were downloaded from the TCGA database, and they were derived from different organs or tissues, including connective and subcutaneous soft tissues, the retroperitoneum and peritoneum, uterus, nose, bones, joints and articular cartilage of the limbs. Unfortunately, we did not find information that could divide the sarcoma samples into fibrosarcoma, osteosarcoma, and other subtypes, in which the effects of SIX1 may be not identical. From the results, we hypothesized that SIX1 may suppress antitumor immune responses in the TME through upregulation of collagen genes. As SIX1 regulates more than ten collagen genes (Fig. 4C), it was difficult to study these collagens simultaneously. RNA-seq data analysis showed that collagen VI was the most highly expressed collagen in MCA205 cells (Fig. S5A). Therefore. We chose collagen VI as a representative molecule to explore whether collagens expressed in cancer cells play an important immunosuppressive function. As a proof of principle, we established a *Col6a1* knockout MCA205 cell line and found that *Col6a1* deficiency in cancer cells significantly increased cytokine secretion and immune cell infiltration (Fig. 5I, J). These results suggested that COL6A1 might play an important role in suppressing antitumor immune effects in the TME. Moreover, rescued expression of *Col6a1* in *Six1*^−/−^ MCA205 cancer cells was sufficient to abolish the CD8^+^ T cell infiltration induced by *Six1* deletion (Fig. S5F). It should be noted that further studies are required to determine whether other SIX1-regulated collagen family members also play similar roles and whether *Six1* deficiency ultimately induces antitumor immune responses via LAIRs.

Mechanistically, it has been reported that SIX1 can regulate TGFBR1 and TGFB signaling [51, 52,]. SIX1 acts as a transcription factor, and the JASPAR CORE database revealed that the SIX1 binding DNA motif contains a TATCA sequence (Fig. 6J). After DNA sequence alignment, we found that the *Tgfbr2* gene contained two putative SIX1-binding sites located at positions −240 and −664. A dual-luciferase reporter assay (Fig. 6J) and ChIP-qPCR (Fig. S6B) confirmed that *Six1* may bind to the *Tgfbr2* promoter region at position −240 and activate the transcription of the *Tgfbr2* gene. In addition, rescued expression of *Tgfbr2* in *Six1*^−/−^ MCA205 cancer cells restored collagen VI gene expression (Fig. 6H). Our studies therefore indicate that SIX1 may activate the TGF-β pathway by directly binding to the *Tgfbr2* promotor, which can subsequently promote collagen expression. Recent reports have shown that the TGF-β pathway shapes the TME to restrain antitumor immunity by restricting T cell infiltration [28]. Overall, our studies, combined with the literature, suggest a potential working model of SIX1-mediated tumor growth. SIX1 increases the expression of collagen genes via the TGF-β pathway. Upregulated collagens further suppress antitumor immunity by restricting immune cells infiltration and inhibiting CD8^+^ T cells activation (Fig. 6K).

Collagen has been studied broadly as a potential therapeutic target because it is upregulated in various human solid tumors and because it has pro-tumor cells survival activity in conferring resistance to radiation, chemotherapies, and immunotherapies [53]. As an exploratory study, our results have shown that loss of *Six1* in cancer cells can induce long-lasting antitumor immune responses. It is possible to turn “cold” tumors into “hot” tumors by inhibiting the expression of SIX1. However, the caveat that the human immune system may be different from the mouse immune system remains, and whether our findings in mice can be directly applied to humans requires further investigation. Nevertheless, because of the fibrotic nature of many solid cancers and the increased levels of SIX1 in tumor tissue, the identification of SIX1 as a master regulator of collagens and a novel suppressor of antitumor immunity may help us to understand how cancer cells control the TME and to design novel cancer immunotherapies.

## Materials and methods

### Raw data acquisition and preprocessing

The mRNA expression profiles and clinical information of 260 sarcoma (SARC) tumor samples and 521 colon adenocarcinoma (COAD) samples (tumor = 480, paracancerous controls = 41) were downloaded from the UCSC Xena database. Two hundred normal muscle tissue samples with RNA-seq data were obtained from the GTEx database (https://xenabrowser.net/). Before further comparison, the “scale” function in the “limma” package (version 3.6.3) was applied to normalize the data among the databases.

### Construction of a prognostic model

The univariate Cox regression method was used to identify Six1 expression with potential prognostic significance (Table 1). All *p* values were adjusted with the Benjamini–Hochberg correction algorithm. We divided the patients into two groups based on the median Six1 expression level. Statistical significance was tested via the log-rank test with the significance threshold for the *p* value set as 0.05. To evaluate the performance of the prognostic signature in SARC samples with Six1 expression data, time-dependent ROC curves were generated using the R package survival ROC.

### Identification of DEGs in tumor and normal samples

Data processing was performed by using the R Bioconductor (version 3.6.3) package. Normalization of data in the TCGA and GTEx datasets was conducted using the “normalize between array” function of the “limma” package. The DEGs between the SARC samples and normal controls were identified using the “limma” package. Genes with a log2 |fold change | >1 and false discovery rate (FDR) < 0.05 were considered DEGs. There were 3523 upregulated and 4172 downregulated mRNA transcripts in SARC and 1123 upregulated and 939 downregulated mRNA transcripts in COAD.

### Analysis of tumor-infiltrating immune cells (TICs)

The relative abundance of TICs in SARC samples with different Six1 expression statuses was calculated with CIBERSORT (https://cibesort.stanford.edu/). Only samples with a *p* value < 0.05 were selected for follow-up analyses.

### Cell culture

The MCA205 murine fibrosarcoma cell line, TC1 murine lung epithelial cell line, MC38 colon cancer line and HEK293 human embryonic kidney cell line were maintained in Dulbecco’s modified Eagle’s medium (DMEM) supplemented with 10% fetal bovine serum (Gibco) and 1% penicillin/streptomycin (Gibco). All cell lines were cultured in a humidified chamber with 5% CO2.

### Construction of stable cell lines with the CRISPR/Cas9 system

Single guide RNA (sgRNA) oligonucleotides targeting mouse *Six1*, *Tgfbr2*, or *Col6a1* were synthesized and cloned into the LentiCRISPR v2 vector (Addgene #52961). Three plasmids including pMD2.G (Addgene #12259), psPAX2 (Addgene #12260), and LentiCRISPR v2 or a control vector were cotransfected into HEK293 cells using Lipofectamine 3000 (Thermo Fisher Scientific) for 48 h. Viral stocks were collected and used to infect target cells. Beginning at 48 h postinfection, cells were cultured in puromycin (4 μg/mL, InvivoGen, cat# ant-pr-1) for at least 7 days. Monoclonal cells acquired with a FACSAria™ III cell sorter (Becton Dickinson, San José, CA, USA) were cultured in a 96-well plate. The sequences synthesized in this study are listed in Supplementary Table S1.

### Tumor models

All animal studies were reviewed and approved by the Institutional Animal Care and Use Committee of the Suzhou Institute of Systems Medicine. Female C57BL/6 and athymic nude BALB/c mice (nu/nu) (6–8 weeks) were purchased from Beijing Vital River Company. Mice were randomly divided into the indicated groups (5 mice/group) before inoculation. Cancer cells (2 × 10^6^ cells in 100 μl PBS per mouse) were subcutaneously implanted. Tumor size was monitored using calipers 2–3 times per week and calculated by multiplying the length by the width. In some experiments, CD8^+^ T cells were depleted by injecting 200 µg/mouse anti-CD8 antibodies (BE0004-1, BioXCell) intravenously at the indicated time points. Tumors were harvested on day 7–12 postimplantation for RNA sequencing, flow cytometric analysis, immunofluorescence staining, and ELISpot analysis. Tumor growth curves are depicted with the error bars indicating the mean ± SD at each time point. Cancer cells were injected via the tail vein, and mortality was recorded every 24 h. Kaplan–Meier survival curves were also plotted. Animals were euthanized with CO_2_ when the tumor volume reached 300 mm^2^.

### RNA sequencing

Cells and tumor tissues were lysed directly by grinding, and total RNA was extracted using the RNeasy Mini Kit (QIAGEN, cat# 74104). Six hundred nanograms of total RNA was used for reverse transcription into cDNA with ProtoScript II Reverse Transcriptase (New England BioLabs, cat# E7420L). The double-stranded cDNA was purified with Agencourt AMPure XP Beads (Beckman, cat# A63881) and then ligated with paired-end adaptors by Multiplex Oligos for RNA sequencing (GSE183580). Sequencing was performed with Illumina HiSeq X 10, and data were analyzed based on the Linux system. The expression profile of DEGs was identified by the R language, including the “edgeR” and “gplots” packages.

### Gene set enrichment analysis (GSEA)

GSEA was performed using GSEA 4.1.0 software according to the guidance of the official website. The entire normalized RNA expression count matrix, without limiting the input to only DEGs, was taken as the input. The expression count matrix was divided into two groups: (1) the KO-High group and (2) the WT-Low group. Hallmarks were selected from the gene set database, and a number of permutations were conducted 1000 times according to default weighted enrichment statistics.

### RNA extraction and RT–qPCR

According to the manufacturer’s instructions, cell pellets were collected and then subjected to total RNA extraction using NucleoZol (MNG, Cat# 740404.200). The extracted RNA was reverse transcribed into cDNA using a One Step PrimeScript RT–PCR kit (TaKaRa, Cat# 6110A) and used for RT–qPCR. Gene-specific primers with the sequences listed in Table S2 and SYRB Green qPCR mix (Bimake. Cn, Cat# B21202) were used for PCR amplification and detection on the Light Cycler Real-Time PCR System (Roche). RT–qPCR data were normalized to GAPDH and are presented as the fold change in gene expression in the test sample compared to the control.

### Protein extraction and western blot analysis

Cells were lysed in lysis buffer, and the total protein concentration was determined with the BCA Protein Assay Kit (Beyotime, cat# P0011). Equal amounts of protein samples were electrophoresed by SDS–PAGE and then transferred to PVDF nitrocellulose membranes. The membranes were blocked in 5% BSA at room temperature for 1 h and incubated with the following specific primary antibodies: anti-SIX1 (1:1000, CST, cat# 16960), anti-collagen VI (1:1000, Abcam, cat# ab182744), anti-TGF beta receptor II (1:1000, Abcam, cat# ab269279), anti-Smad2/3 (1:1000, CST, cat# 5678), anti-phospho-Smad2 (Ser465/467)/Smad3 (Ser423/425) (1:1000, CST, cat# 8828), and anti-β-ACTIN (1:1000, CST, cat# 4970). The next day, the membranes were washed with TBST three times and incubated with an anti-rabbit IgG HRP-linked antibody (1:2000, CST, cat# 7074) at room temperature for 50 min. The membranes were imaged using a ChemiDoc XRS + system (Bio–Rad, USA). To further confirm the involvement of TGF-β signaling in mechanical SIX1-induced collagen expression, cells were pretreated with or without mTGF-β1 (50 ng/ml, CST, cat# 5231LF) for 2 or 4 h.

### Colony formation assay

Cells (1000 per well) were plated in six-well plates. After culturing for 10 days, the cells were fixed with 4% PFA for 15 min and stained with a 0.5% crystal violet solution for 30 min.

### Flow cytometry

Mice were humanely euthanized, and mouse tumors were harvested. Whole tumors were cut and minced into small pieces, followed by digestion in Liberase TL Research Grade 10 (2 µg/mL, Roche, 05401020001) and DNase I (Sigma, 260913-10 MU) in DMEM at 37 °C for 30 min. The digested tissue was filtered through a 70-mm cell strainer (Thermo Fisher Scientific). Cell suspensions were stimulated with PMA (100 µg/mL) plus the protein transport inhibitors Golgi stop and Golgi plug (1:1000, BD Bioscience, 51-2301 KZ) at 37 °C. After 4 h, live cells were identified by vivid yellow staining (Invitrogen, cat# L34959). Cells were stained for cell surface markers including CD45.2 (1:100, BioLegend, 109814), CD8 (1:100, BioLegend, cat# 100708), CD11c (1:100, BioLegend, 117311), and IA/IE (1:100, BioLegend, 107608) at 4 °C for 30 min. For intracellular staining, cells were fixed and permeabilized with a fixation/permeabilization kit (BD Bioscience, 554714) for 30 min at 4 °C and then stained with anti-IFN-γ (1:100, BioLegend, 505813) and anti-granzyme B (1:100, BioLegend, 515406) antibodies. Cells were imaged on a BD LSRFortessa (BD Biosciences) and analyzed using FlowJo software (TreeStar).

### Immunofluorescence staining and imaging

Immunofluorescence staining was used to analyze 4% paraformaldehyde-fixed, 0.1% Triton X-100-permeabilized WT MCA205 and *Six1*^−/−^ MCA205 cells labeled with an anti-collagen VI antibody (1:200, Abcam, cat# ab182744) at 4 °C overnight. After washing three times, the cells were incubated with an Alexa Fluor Plus 555-conjugated (1:500, Invitrogen, A32732) secondary antibody for 1 h. Cell nuclei were counterstained with Hoechst 33258 (Thermo Fisher Scientific, H3569) for 5 min at RT. Images were acquired using a confocal microscope (Leica TCS SP8).

Tumors were harvested on day 8 or 12, fixed in 4% paraformaldehyde for 24 h, dehydrated in a 30% (wt/vol) sucrose solution for 48 h and finally embedded in OCT at room temperature. Four-micrometer frozen sections of tumor tissues were obtained and fixed in ice-cold acetone for 15 min. After blocking with 5% goat serum in PBS for 1 h, the tumor tissue sections were incubated with primary antibodies against CD8a (1:100, Abcam, ab217344) at 4 °C overnight. After washing three times, the tumor tissue sections were incubated with an Alexa Fluor Plus 555-conjugated (1:500, Invitrogen, A32732) secondary antibody for 1 h. Cell nuclei were counterstained with Hoechst 33258 (Thermo Fisher Scientific, H3569) for 5 min at RT. Images were acquired using a confocal microscope (Leica TCS SP8) and analyzed with ImageJ software.

### Immunohistochemical (IHC) analysis

Paraffin-embedded tumor tissue slides were labeled with an anti-collagen VI antibody (1:250, Abcam, cat# ab182744). Next, the slides were incubated with a goat anti-rabbit IgG antibody labeled with HRP (Shanghai Gene Company, GK500705) for 40 min, stained with DAB substrate for 2–10 min and counterstained with hematoxylin.

### Enzyme-linked immunospot (ELISpot) assay

According to the manufacturer’s instructions, IFN-γ secretion was measured with BD ELISpot assay kits (BD Biosciences, 551881). Briefly, tumors were sterilely harvested and processed into single-cell suspensions. Cells were seeded at 2 × 10^6^ cells per well in a capture antibody-precoated ELISpot plate and then cultured in a humidified incubator with 5% CO_2_ at 37 °C for 20 h. Subsequently, the cells were removed, and the plate was washed three times. The production of IFN-γ was measured by adding a detection antibody for 2 h at RT, followed by washing three times and incubation with an HRP-linked secondary antibody for 2 h at RT. Finally, 100 µL final substrate solution (AEC) was used for color development. Red dots were observed using CTL ImmunoSpot^®^ S6 Analyzers (LLC, OH, USA).

### Dual-luciferase reporter assay

The primers containing XhoI and HindIII sites that were used to amplify the promoter of *Tgfbr2* gene are shown in Table S3. Amplified products were cloned into the luciferase reporter vector pGL4.0 (Addgene, 84924). The *Six1* coding DNA sequence was cloned into dCAS9-VP64-GFP (Addgene, 61422). MCA205 WT cells were seeded in 24-well plates (2 × 10^5^/well) and incubated overnight before transfection. Then, pGL4-Tgfbr2, Renilla luciferase plasmids and SIX1 plasmids or empty plasmids were cotransfected into the cells by using Lipofectamine 3000 (Invitrogen, L3000-015). After 24 h, firefly and Renilla luciferase activities were detected using a dual-luciferase reporter system (Promega, E1960). The efficacy was calculated as the ratio of firefly luciferase activity/Renilla luciferase activity.

### Chromatin immunoprecipitation (ChIP) analysis

ChIP analysis was performed by using the SimpleChIP^®^ Plus Enzymatic Chromatin IP Kit (CST, cat# 9005s) according to the manufacturer’s manual. Since an anti-SIX1 antibody cannot be used for ChIP analysis, we selected a SIX1-Flag-expressing stable MCA205 cell line using a lentivector constructed based on the dCAS9-VP64-GFP vector (Addgene, 61422). Briefly, 37% formaldehyde (final concentration 1%) was incubated with a SIX1-Flag-MCA205 cell culture to cross-link protein with DNA for 10 min, and the reaction was then stopped with a glycine solution for 5 min. The cells were washed twice with ice-cold PBS, lysed in Membrane Extraction Buffer for 10 min, and centrifuged at 2000 *g* for 5 min to collect the nuclei. The nuclei were resuspended in 100 µl Digestion Buffer and then digested by incubation with micrococcal nuclease at 37 °C for 20 min. The reaction was terminated with 0.5 M EDTA. The nuclei were precipitated by centrifugation at 16,000 *g* for 1 min, resuspended in 100 µl Chip Buffer, and then sonicated (three 20-s pulses at 20 W for 4 × 10^6^ cells) to break the nuclear membrane. Following centrifugation at 9400 *g* for 10 min, the supernatant was collected, and chromatin digestion and concentration were analyzed. For optimal ChIP results, ~10 µg digested, cross-linked chromatin was used per immunoprecipitation. Diluted digested chromatin was incubated overnight with an anti-Flag antibody (CST, #14793 s) or normal IgG antibody control (CST, 2729 s). Then, 30 µL ChIP-grade Protein G magnetic beads were added and incubated for 2 h at 4 °C with rotation. The beads were collected, washed sequentially with a low salt wash and high salt wash and then resuspended in 150 µl 1X ChIP Elution Buffer for 30 min at 65 °C with gentle vortexing. The protein-DNA cross-links were reversed with 5 M NaCl and Proteinase K. The pulled-down DNA was purified with a spin column and used for qPCR. The primers used for the detection of *Tgfbr2* promoter regions that contain the predicted SIX1-binding sequence are listed in Table S4. For each qPCR assay, triplicate samples were used, and data were normalized to the respective input samples.

### Statistics

Statistical analyses were performed with GraphPad Prism 7 software. Continuous variables are presented as the mean ± SD. Data with a normal distribution were analyzed by one-way ANOVA or unpaired two-tailed Student’s *t*-tests, and tumor growth curves were compared by the Mann–Whitney *U* test or two-way ANOVA. *P* values are indicated as * P < 0.05, ** P < 0.01, and *** P < 0.001.

## Supplementary information

Figure S1

Figure S2

Figure S3

Figure S4

Figure S5

Figure S6

Supplementary Table

Supplementary Figures
