# Supplementary figures and images for: Homeoprotein SIX1 compromises antitumor immunity through TGF-β-mediated regulation of collagens
Source: Cell Mol Immunol. 2021 Nov 15;18(12):2660–72. doi: 10.1038/s41423-021-00800-x (PMC8633173; doi:10.1038/s41423-021-00800-x)

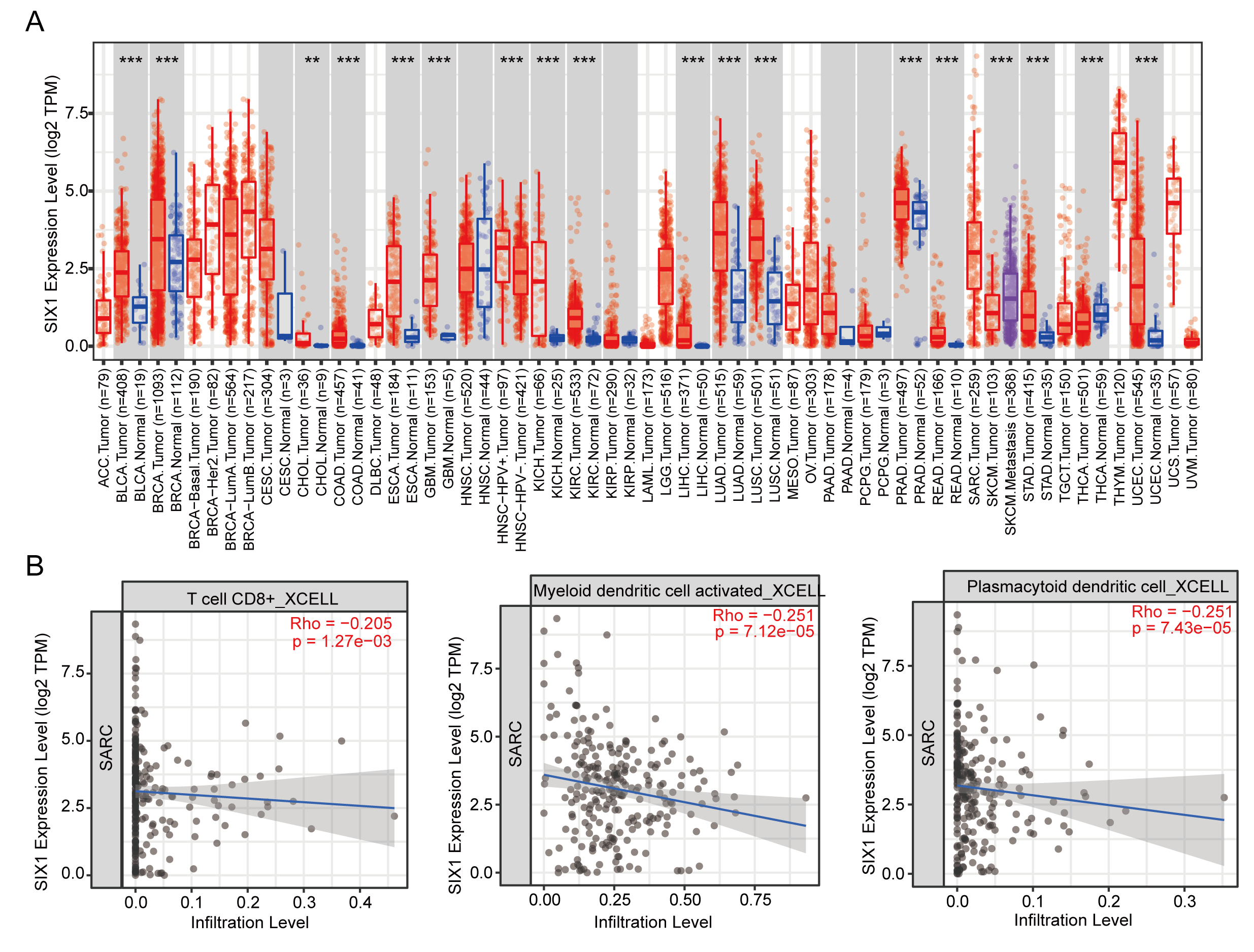

Supplement: Supplementary file 1 — Figure S1 [file 41423_2021_800_MOESM1_ESM.tif]

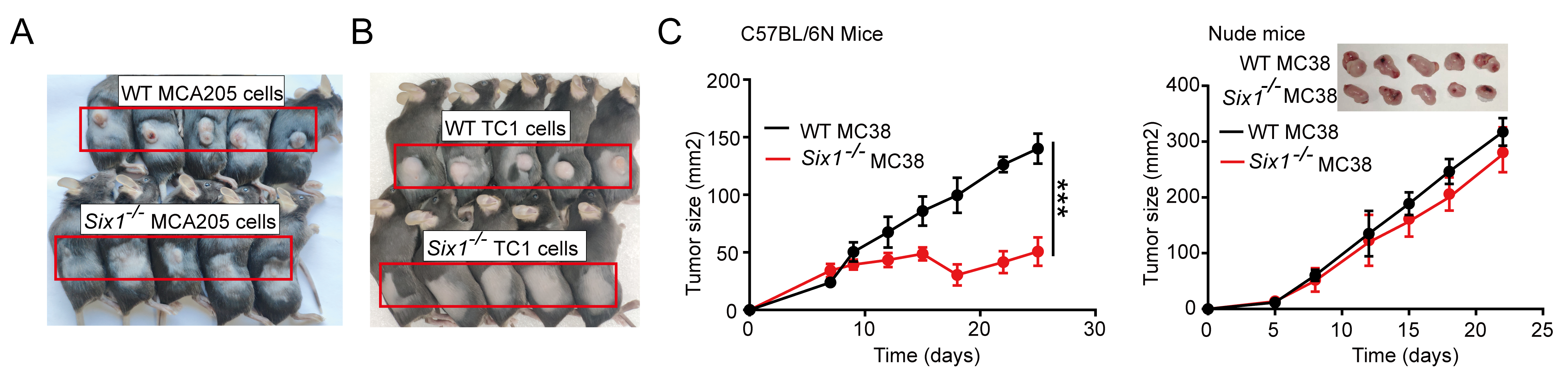

Supplement: Supplementary file 2 — Figure S2 [file 41423_2021_800_MOESM2_ESM.tif]

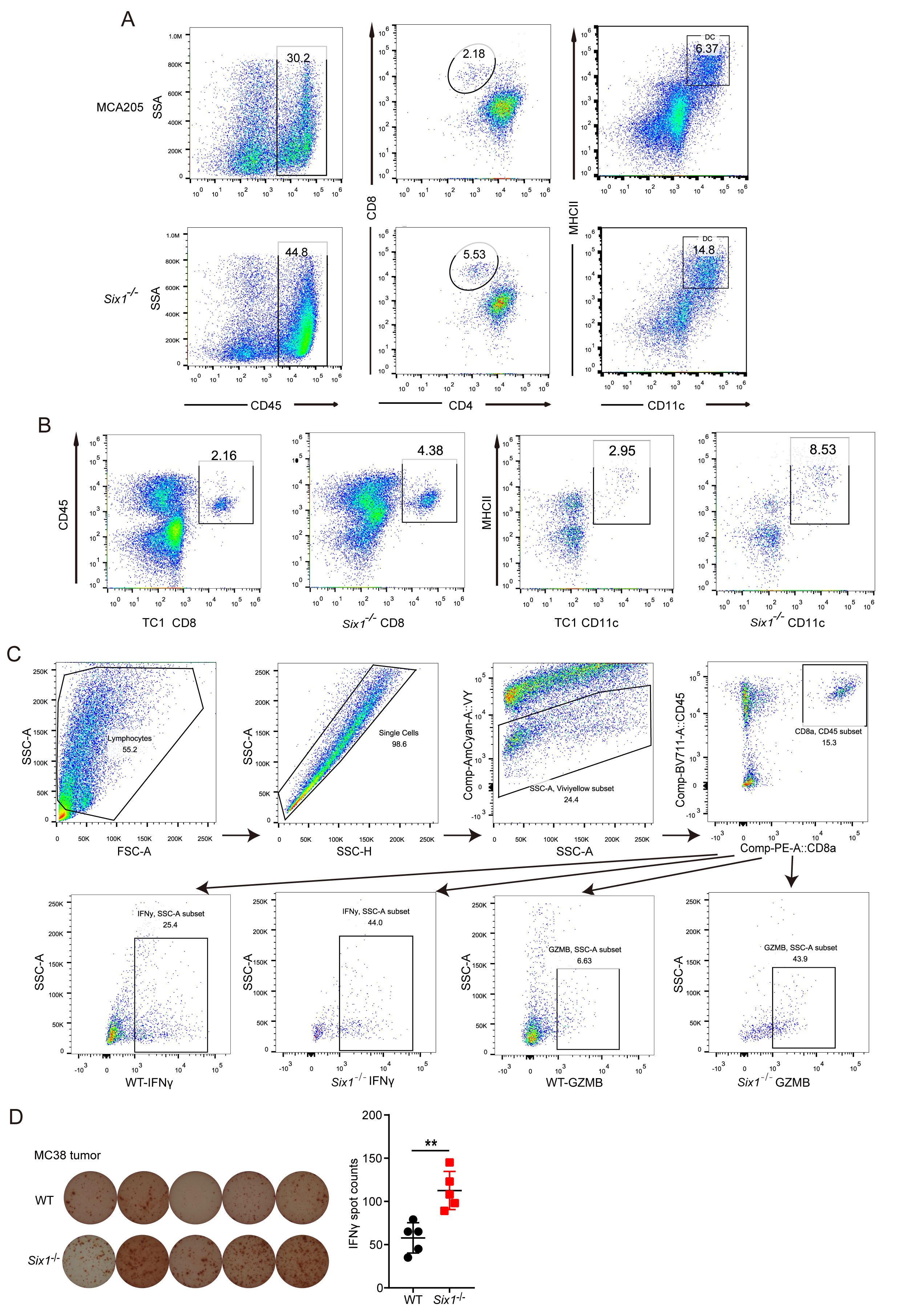

Supplement: Supplementary file 3 — Figure S3 [file 41423_2021_800_MOESM3_ESM.tif]

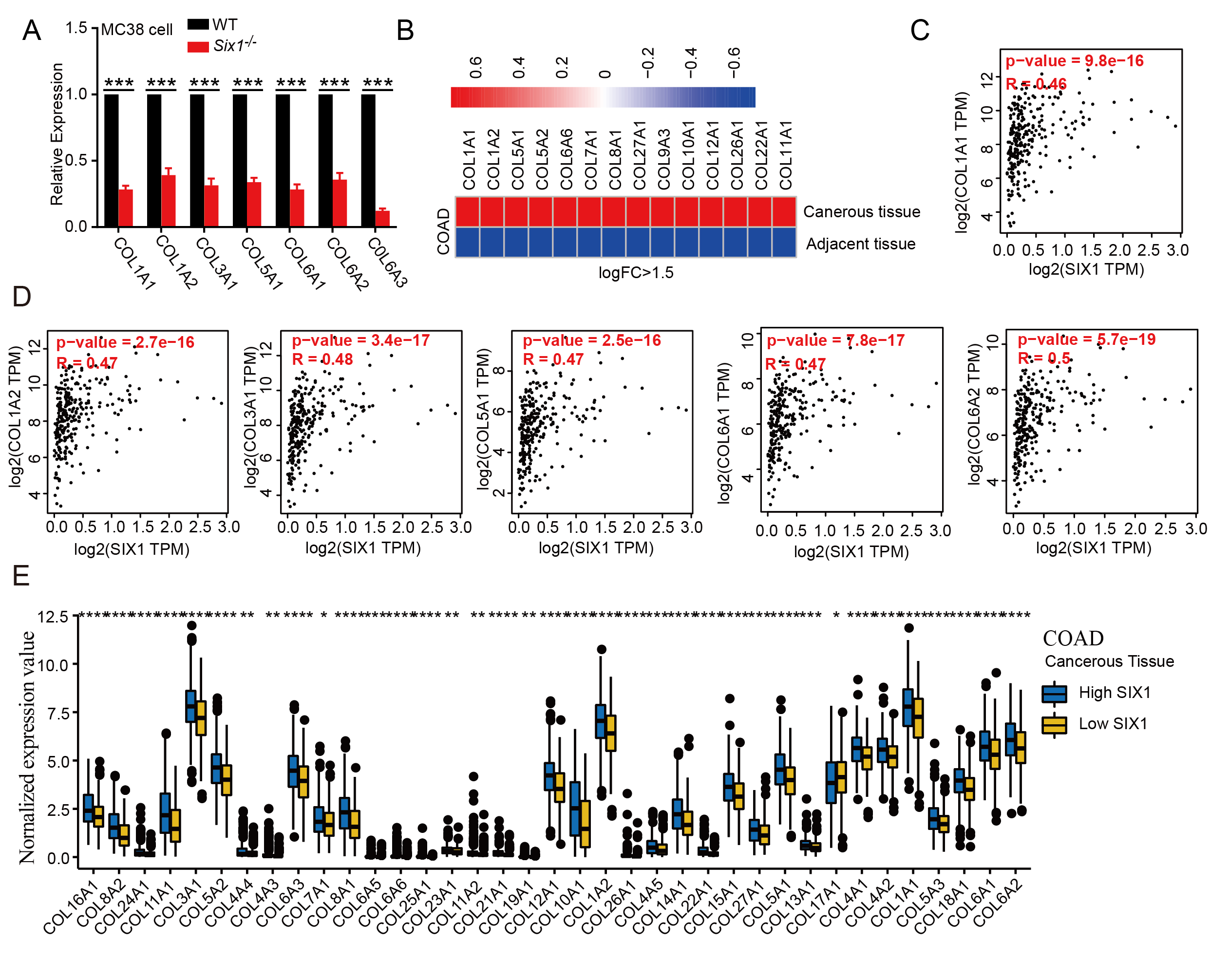

Supplement: Supplementary file 4 — Figure S4 [file 41423_2021_800_MOESM4_ESM.tif]

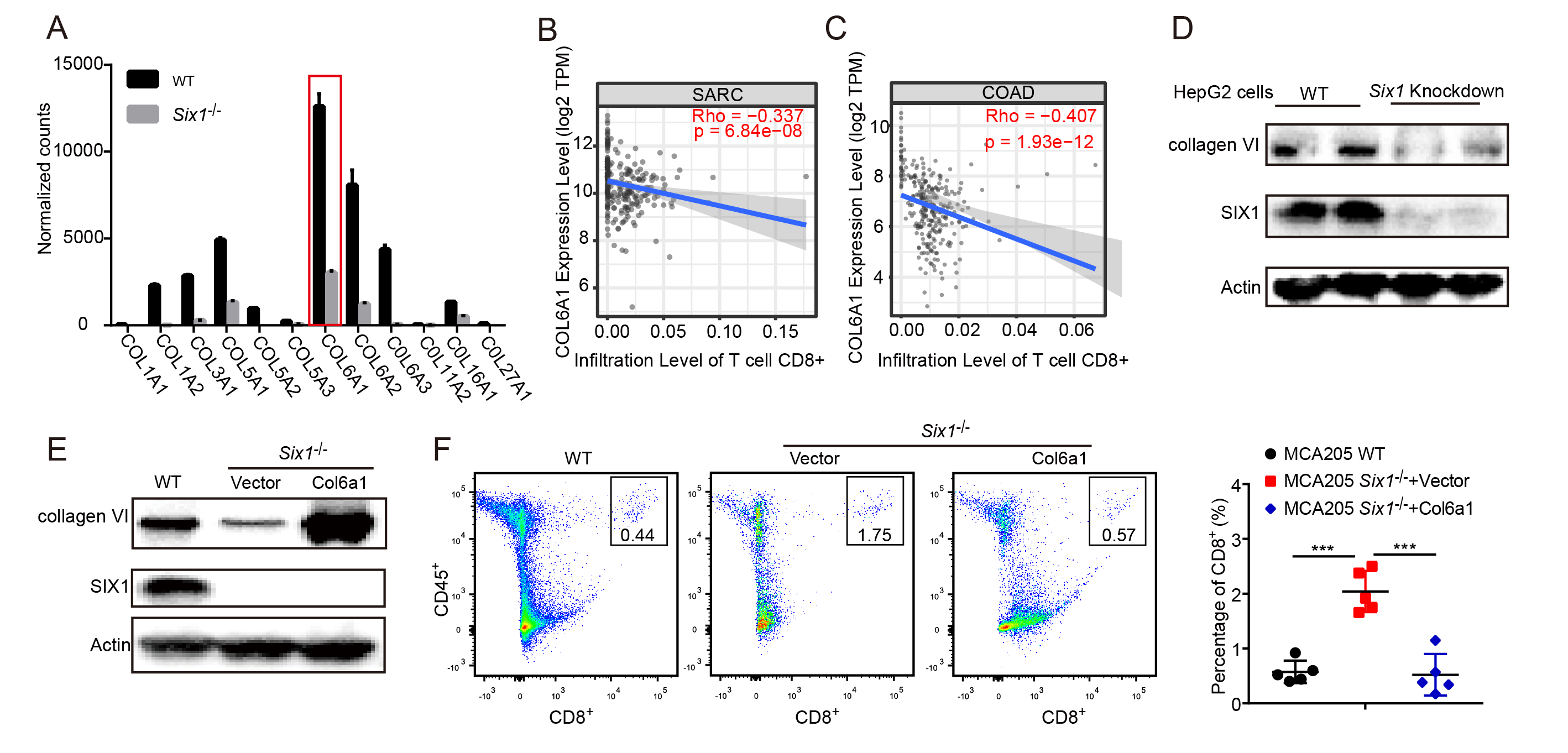

Supplement: Supplementary file 5 — Figure S5 [file 41423_2021_800_MOESM5_ESM.tif]

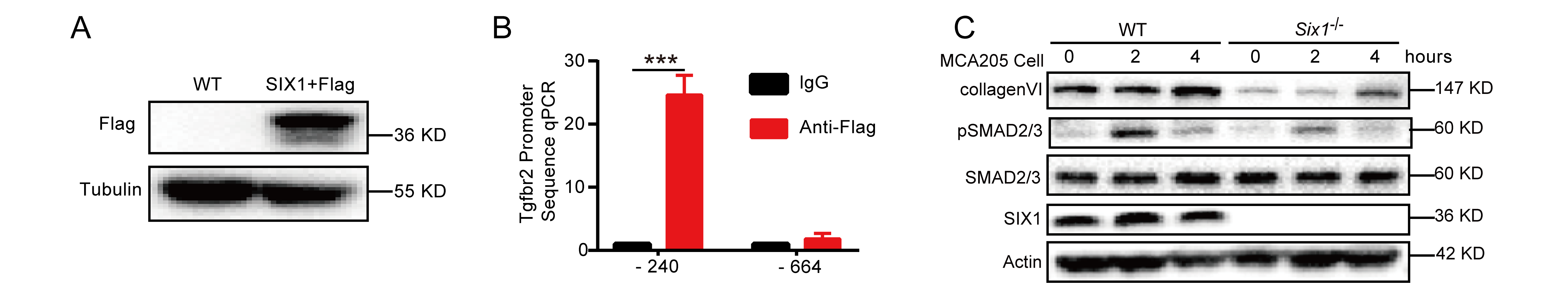

Supplement: Supplementary file 6 — Figure S6 [file 41423_2021_800_MOESM6_ESM.tif]
