# Supplementary material for: Homeoprotein SIX1 compromises antitumor immunity through TGF-β-mediated regulation of collagens
Source: Cell Mol Immunol. 2021 Nov 15;18(12):2660–72. doi: 10.1038/s41423-021-00800-x (PMC8633173; doi:10.1038/s41423-021-00800-x)
Supplement: Supplementary file 7 — Supplementary Table [file 41423_2021_800_MOESM7_ESM.doc]

Table S1. Sequence of Guide RNA (sgRNA) oligonucleotides.

| Gene | Forward | Reverse |
| --- | --- | --- |
| *Six1* | CACCGGGGTCGGCCGCGAAGTTTCT | AAACAGAAACTTCGCGGCCGACCC |
| *Tgfbr2* | CACCGCCACGCGAAGGGCAACCTGC | AAACGCAGGTTGCCCTTCGCGTGGC |
| *Col6a1* | CACCGGCATAGCCGCGATGCAGAAG | AAACCTTCTGCATCGCGGCTATGCC |

Table S2. Primers designed for real-time PCR.

| Gene | Forward | Reverse |
| --- | --- | --- |
| *Gapdh* | AGGTCGGTGTGAACGGATTTG | TGTAGACCATGTAGTTGAGGTCA |
| *Col1a1* | CTGGCGGTTCAGGTCCAAT | TTCCAGGCAATCCACGAGC |
| *Col1a2* | AAGGGTGCTACTGGACTCCC | TTGTTACCGGATTCTCCTTTGG |
| *Col3a1* | ACGTAGATGAATTGGGATGCAG | GGGTTGGGGCAGTCTAGTG |
| *Col5a2* | ACAGGTGAAGTGGGATTCTCA | CCATAGCACCCATTGGACCA |
| *Col6a1* | CTGCTGCTACAAGCCTGCT | CCCCATAAGGTTTCAGCCTCA |
| *Col6a2* | GCTCCTGATTGGGGGACTCT | CCAACACGAAATACACGTTGAC |
| *Col6a3* | AAGGACCGTTTCCTGCTTGTT | GGTATGTGGGTTTCCGTTGAG |
| *Tgfbr2* | TTGGATTGCCAGTGCTAACCC | AACAAGCCACAGTAACATGACA |
| *Gzma* | TGCTGCCCACTGTAACGTG | GGTAGGTGAAGGATAGCCACAT |
| *GzmB* | CCACTCTCGACCCTACATGG | GGCCCCCAAAGTGACATTTATT |
| *Cxcl9* | TCCTTTTGGGCATCATCTTCC | TTTGTAGTGGATCGTGCCTCG |
| *Cxcl10* | CCAAGTGCTGCCGTCATTTTC | GGCTCGCAGGGATGATTTCAA |
| *Cd8a* | CCGTTGACCCGCTTTCTGT | CGGCGTCCATTTTCTTTGGAA |
| *Ifng* | ATGAACGCTACACACTGCATC | CCATCCTTTTGCCAGTTCCTC |

Table S3. Primers of Tgfbr2 promoter.

|  | Forward | Reverse |
| --- | --- | --- |
| motif A | AGCCTCGAGAGCTTCACCCAGCAAACCACGCCCA | GCCAAGCTTTCGTCGGTCGGTGCGCGCGAG |
| motif B | AGCCTCGAGGCCCTGAACTCACAGAGACCCGCCCAC | GCCAAGCTTCCAGGCACCCCCACACCTTCTAGGCA |

Table S4. Primers designed for Chip-qPCR.

|  | Forward | Reverse |
| --- | --- | --- |
| position –240 | ACGTGTTCGGGACTCTGAAGC | GCCAAGTCTGGGCTCCAAGT |
| Position -664 | ATCATCGTCTGGCCCCAAACAT | ATCATCGTCTGGCCCCAAACAT |
