# Supplementary material for: Homeoprotein SIX1 compromises antitumor immunity through TGF-β-mediated regulation of collagens
Source: Cell Mol Immunol. 2021 Nov 15;18(12):2660–72. doi: 10.1038/s41423-021-00800-x (PMC8633173; doi:10.1038/s41423-021-00800-x)
Supplement: Supplementary file 8 — Supplementary Figures [file 41423_2021_800_MOESM8_ESM.docx]

**Figure S1.**

**High expression of Six1 negatively correlates with immune cell infiltration in the TME.**

(A) Six1 expression between tumor and adjacent normal tissues across all TCGA tumors from the Gene_DE of Exploration module in TIMER2.0. The distributions of gene expression levels are displayed using box plots. Statistical significance results computed by the Wilcoxon test were annotated with stars (**p value < 0.05*; ***p value <0.01*; ****p value <0.001*).

(B) Relevance of Six1 expression to the immune infiltration in SARC patients from the Gene of immune module in TIMER2.0. The expression of Six1 was significantly negatively correlated with the levels of CD8 T cell and DC infiltration in tumor-bearing patients.

**Figure S2. Immune-dependent mechanisms are responsible for the reduced tumor growth of *Six1-*deficient cancer cells.**

(A) Deletion of *Six1* in MCA205 tumor cells inhibited tumor growth *in vivo*. The gross appearance of C57BL/6N mice subcutaneously transplanted with 2*10^6^ *Six1^-/-^* or WT MCA205 tumor cells was examined.

(B) Deletion of *Six1* in TC1 tumor cells inhibited tumor growth *in vivo*. The gross appearance of C57BL/6N mice subcutaneously transplanted with 2*10^6^ *Six1^-/-^* or WT TC1 tumor cells was examined.

(C) Tumor growth curves for C57BL/6N and nude mice inoculated with MC38 cells. C57BL/6N or nude mice were subcutaneously injected with 2*10^6^ *Six1^-/-^* or WT MC38 cells (n=5 per group). Tumors were evaluated with calipers two or three times per week starting on Day 5 post inoculation. Data are presented as the mean ± SD at each time point. Statistical significance was evaluated with the Mann–Whitney U test; ****p < 0.001*.

**Figure S3. *Six1* deficiency triggers cellular immune responses *in vivo.***

(A) Representative scatterplots of the gated immune cells in Figure 3I are shown. A total of 2*10^6^ *Six1^-/-^* or WT MCA205 tumor cells were subcutaneously transplanted into the back flank of C57BL/6N mice. Eight days after transplantation, tumor tissues were collected and subjected to flow cytometric analysis.

(B) Representative scatterplots of the gated immune cells in Figure 3J are shown. A total of 2*10^6^ *Six1^-/-^* or WT TC1 tumor cells were subcutaneously transplanted into the back flank of C57BL/6N mice. Eight days after transplantation, tumors were collected and subjected to flow cytometric analysis.

(C) Representative scatterplots of the gated immune cells in Figure 3M are shown. A total of 2*10^6^ *Six1^-/-^* or WT MCA205 tumor cells were subcutaneously transplanted into the back flank of C57BL/6N mice. Eight days after transplantation, tumors were collected and subjected to flow cytometric analysis. CD8+ T cell gating was performed after background assessment. The numbers indicate the percentages of GZMB- and IFN-γ-positive cells in the gated CD8^+^ T cell population.

(D) ELISpot assay assessing the secretion of IFN-γ in MC38 tumor tissue. A total of 2*10^6^ *Six1^-/-^* or WT MC38 tumor cells were subcutaneously transplanted into the back flank of C57BL/6N mice. The tumors were subjected to ELISpot analysis. The number of spots was enumerated on an ELISpot reader, and the results are presented as spot-forming units. Data are shown as the mean ± SD; ***p < 0.01,* unpaired Student’s-test.

**Figure S4. SIX1 promotes the expression of collagen genes.**

(A) RT–qPCR analysis of the mRNA expression levels of collagen genes between *Six1^-/-^* and WT MC38 cells. GAPDH was used as a loading control; ****p < 0.001*, unpaired Student’s t-test.

(B) Heatmap depicting differential expression of collagen genes between adjacent and cancerous tissue in COAD data downloaded from the TCGA.

(C and D) Correlations between Six1 and Col1a1 or Col6a1 transcripts in COAD cancer tissues were analyzed using GEPIA (http://gepia.cancer-pku.cn).

(E) Comparison of the expression levels of collagen genes between high and low Six1 expression groups of COAD samples obtained from the TCGA.

**Figure S5. Reduced tumor growth of *Col6a1*-deficient cancer cells correlated with enhanced immune cell infiltration.**

(A) Normalized counts of differentially expressed collagen genes from RNA-seq data comparing WT and *Six1^-/-^* MCA205 cells.

(B) Relevance of Col6a1 expression to the immune infiltration in SARC patients from the Gene of immune module in TIMER2.0. The expression of Col6a1 was significantly negatively correlated with the levels of CD8^+^ T cell infiltration in the tumor patients.

(C) Relevance of Col6a1 expression to the immune infiltration in COAD patients from the Gene of immune module in TIMER2.0. The expression of COL6A1 was significantly negatively correlated with the levels of CD8^+^ T cell infiltration in the tumor patients.

(D) Western blot analysis of COL6A1 and SIX1 in matched WT and *Six1-*knockdown HepG2 cells. β-Actin was used as a loading control.

(E) Western blot analysis of SIX1 and collagen VI in matched WT, *Six1^-/-^* and *Col6a1*-overexpressing *Six1^-/-^* MCA205 cells.

(F) Profiling of immune cells (defined by specific markers) in the TME by flow cytometry. A total of 2*10^6^ WT, *Six1^-/-^* or *Col6a1*-overexpressing *Six1^-/-^* MCA205 cells were subcutaneously transplanted into the back flank of C57BL/6N mice. On Day 8, tumor tissues were subjected to flow cytometric analysis. Data are shown as the mean ± SD; n=5. ***p < 0.01, ***p < 0.001*, unpaired Student’s-test.

**Figure S6. SIX1 binds to the *Tgfbr2* promoter.**

(A) Western blot analysis of SIX1-flag in MCA205 cells. Tubulin was used as a loading control.

(B) ChIP-qPCR. MCA205 cells were transfected with a SIX1-Flag-expressing vector. ChIP was performed using an anti-flag antibody. One of three similar experiments was performed to detect different SIX1-binding regions in the *Tgfbr2* gene promoter, and the data were normalized to the respective input samples. Mean ± SD; n=3 repeats. Statistics: unpaired Student’s-test, ****P<0.001.*

(C) The involvement of TGF-β1 signaling in the process of SIX1-induced collagen VI expression. WT and *Six1*^-/-^ MCA205 cells were treated with mTGF-β1 (50 ng/ml) for 2 or 4 hours, and then western blotting was performed.
